# Adjustments for oral fluid quality and collection methods improve prediction of circulating tetanus antitoxin: Approaches for correcting antibody concentrations detected in a non-invasive specimen

**DOI:** 10.1016/j.vaccine.2020.11.027

**Published:** 2021-01-08

**Authors:** Henri Garrison-Desany, Benard Omondi Ochieng, Maurice R. Odiere, Helen Kuo, Dustin G. Gibson, Joyce Were, E. Wangeci Kagucia, Marcela F. Pasetti, Hani Kim, Mardi Reymann, Katherine O'Brien, Kyla Hayford

**Affiliations:** a International Vaccine Access Center, Johns Hopkins Bloomberg School of Public Health, Baltimore, MD, USA; b Centre for Global Health Research, Kenya Medical Research Institute (KEMRI), Kisumu, Kenya; c Department of International Health, Johns Hopkins Bloomberg School of Public Health, Baltimore, MD, USA; d Center for Vaccine Development and Global Health, University of Maryland School of Medicine, Baltimore, MD, USA; e Global Health, Bill & Melinda Gates Foundation, Seattle, WA, USA

**Keywords:** Tetanus toxoid, Serological surveillance, Serological survey, Oral fluid, Serum, Total IgG

## Abstract

**Objectives:**

To examine whether anti-tetanus toxoid (anti-TT) immunoglobulin G (IgG) levels measured in oral fluid and adjusted for collection difficulties and specimen quality are associated with total IgG and anti-TTIgG in oral fluid and assess if statistical adjustment for them improves prediction of anti-TT IgG in serum.

**Methods:**

267 children, ages 12 to 15 months, enrolled in the M-SIMU randomized controlled trial participated in this nested cross-sectional analysis. Venous blood and oral fluid (OF) specimens were collected, and OF collection difficulties such as crying or gagging were recorded. OF volume was documented and total IgG was measured in OF specimens and anti-TT IgG was measured in OF and serum by enzyme immunoassay (EIA). Collection difficulties, volume and sociodemographic characteristics were assessed in relation to total IgG and anti-TT IgG in OF via multivariate regression. These models were extended to evaluate the association between anti-TT IgG in OF and in serum. A prediction model was developed to adjust anti-TT IgG in OF estimates as proxy for serum.

**Results:**

Blood in the specimen, sores in the mouth and crying were positively associated with total IgG concentration while high oral fluid volume and sucking on the swab were inversely associated. None were significant predictors of anti-TT IgG in OF after adjusting for total IgG (geometric mean [GM] ratio: 1.99; 95% confidence interval: 1.78–2.24) and vaccination history (GM ratio: 2.44; 95% CI: 1.98–3.01). When predicting anti-TT IgG levels in serum with OF, total IgG modified the effect of anti-TT IgG in OF.

**Conclusions:**

Anti-TT IgG in OF is a good proxy for levels in serum, after controlling for total IgG in the specimen and other variables. Post hoc adjustments for OF volume and total IgG concentration are an important consideration when conducting serosurveys with oral fluid.

## 1 Introduction

Household surveys with biospecimen collection are increasingly used to assess prevalence of infection [1], [2] and population immunity [3], [4], [5]. Large scale collection of venous blood can be challenging in household surveys due to biosafety risks, the need for skilled phlebotomists, and the need for timely processing and storage of blood specimens [6], [7]. A less invasive specimen, such as oral fluid, may be more acceptable among healthy participants, especially children. Recent studies found parents were less hesitant to enroll their children if studies used less invasive methods of specimen collection such as mouthwashes or cytobrush, and microneedles [8], [9], [10].

Oral fluid (OF), the portion of saliva collected along the gumline and comprised primarily of gingivocrevicular fluid, is rich in plasma-derived immunoglobulins G (IgG) [11], [12]. Thus, OF theoretically reflects the composition of antibodies circulating in blood but tends to be less concentrated. The estimated IgG concentrations of non-oral antigens in OF are highly variable across studies and sampling sites within the oral cavity, ranging from 1:1 to 1:800 compared to concentrations in serum [13], [14], [15], and the total IgG concentration per sample can vary substantially as well. Previous studies found that specific IgG concentrations in OF are influenced by specimen quality including total IgG concentration [16], storage temperature [17], insufficient volume, and food contamination in the OF sample [18].

To further investigate the relationship between antibodies in OF and serum, anti-tetanus toxoid (TT) antibodies can serve as a helpful case study. Anti-TT IgG antibodies above protective levels are only conferred through vaccination [19] with a tetanus antigen-containing vaccine such as pentavalent vaccine (diptheria, tetanus, pertusiss, hepatitis B, and *Haemophilus influenzae* type B). Therefore, infants showing anti-TT IgG are expected to be vaccinated.

Given that serological testing is used increasingly for public health surveillance and research, less invasive methods present an important opportunity to increase participation among different populations [20], [21], [22], [23], [24]. Therefore, validity assessments are a necessary step to address current gaps in our understanding of what impacts discrepancies between serum and OF. While multiple studies report high sensitivity of specific-IgG detection in OF compared to serum [21], [22], [25], [26], [27], [28], other studies found poor sensitivity [3], [21], [29]. It is possible that many causes related to antigen, assay type, collection methods, such as collection device, implementation, handling, and population, such as age and health of participants, may influence accuracy; but, beyond total IgG concentration within a given sample, there is little consensus which factors are most influential, and if they can be mitigated to improve the diagnostic accuracy of OF.

We hypothesize the discrepancies in antibody concentrations between OF and serum are primarily due to variability in the quality and concentration of the OF specimen. Second, we hypothesize the information on specimen collection problems and quality could be measured and used to improve prediction of circulating levels in serum. This study seeks to integrate both factors impacting OF in the field and in the lab using statistical models to provide better estimates of its relationship to that in serum, using anti-TT IgG to illustrate this pathway.

## 2 Methods

### 2.1 Study design

This cross-sectional analysis was nested within the Mobile Solutions for Immunization (M-SIMU study) study, a cluster randomized controlled trial evaluating the impact of reminders and incentives on vaccination coverage [30]. In M-SIMU, children in Nyanza Province of western Kenya were identified within one month of birth. Study staff prospectively documented vaccination at all clinics in the catchment area from birth to 12 months of age and verified through home-based records (such as vaccination cards) and vaccination clinic records. At least two concordant written data sources were required to determine vaccination status. Caregiver recall was not used to assign status with one exception: caregivers who did not show a vaccination card and recalled the child did not receive any pentavalent vaccine doses were classified as unvaccinated. In Kenya, 88.3% of children receive three doses of the pentavalent vaccine by 12 months of age, and 97.0% receive at least one dose of the pentavalent vaccine [31].

Children ages 12 to 14 months of age from the study cohort were eligible for the nested study. Those who were unvaccinated (i.e., received zero pentavalent vaccine doses) or under-vaccinated (i.e., received one or two doses) and a random subset of children who received all three recommended doses by age 12 to 14 months were recruited for participation. OF and venous blood were collected from the child as well as characteristics of the child’s health and problems with specimen collection. Risk factors were grouped into three categories: (1) sociodemographic factors, (2) OF collection problems and child health problems measured in the field, and (3) specimen characteristics measured in the laboratory. Recorded OF collection problems were defined based on those observed in previous studies and revised during pilot testing [18]. Height-for-age and weight-for-age Z-score were calculated according to World Health Organization (WHO) Child Growth Standards [32]. Laboratory data was managed on web-based REDCap software [33], and analyses were conducted using Stata 15 [34].

### 2.2 Specimen collection and testing

Two OF specimens were collected with an Oracol Plus swab (Malvern Medical Developments, Worchester, UK). Each swab was gently rubbed along the both sides of the top gum line for a minimum of 1.5 min, transported in a cool box to the laboratory within 4 h and centrifuged at 1050*g* for 20 min. OF from the two swabs were combined in the lab, and results are reported here in aggregate of both swabs. OF volume was measured, then an equal volume of preservative buffer was added and specimens were stored at −80 °C until testing. The preservative buffer consisted of 0.05% Polyoxyethylene Sorbitan Monolaurate – Tween20 (Sigma #P1379) and 0.01% Chlorhexidine Digluconate (Sigma #C9394). OF specimens were tested for concentrations of total IgG and anti-tetanus toxoid (anti-TT) IgG [35] using in-house indirect enzyme immunoassays (EIA) at the Center for Vaccine Development, University of Maryland following procedures previously described [27].

Up to 3.5 mL of venous blood was collected from children by butterfly needle in BD serum separator vacutainer tubes and left undisturbed for at least 30 min at room temperature. They were carried to the laboratory at 2–8 °C in cool boxes, centrifuged at 1050*g* for 20 min within 8 h of collection, and stored at −80 °C before being shipped for testing at Statens Serum Institut (Copenhagen, Denmark) with an in-house double antigen EIA, methods described elsewhere [36]. Geometric mean concentrations (GMC) of total IgG (mg/L) and anti-TT IgG (IU/mL) were calculated. A threshold of 0.01 IU/mL for serum anti-TT IgG was considered the cutoff for serological immunity.

### 2.3 Risk factor analysis

A directional acyclic graph (DAG) was constructed to theorize the relationship between specimen characteristics and antibody concentrations. Univariate and multivariate regression models were developed to identify risk factors for low total IgG concentration in OF specimens, an indicator of specimen quality. Models were developed for three scenarios of available covariates: (1) sociodemographic (2) field-measured, and (3) laboratory-measured characteristics. Using log_10_-transformed total IgG, candidate models were compared using a Furnival-Wilson leaps-and-bounds algorithm [37] and parsimony, and the best model was selected based on the Akaike’s information criteria (AIC), Bayesian information criteria (BIC) and log likelihood criteria. The geometric mean ratio of total IgG per unit increase in exposure is reported as the outcome, which allows us to generate relative associations while our anti-TT IgG outcome to remains log-transformed in our model. Sensitivity analyses grouped field-measured collection difficulties by their impact on OF IgG: having mouth sores or blood in specimen were considered “blood-related difficulties,” and sucking on swab, dry mouth, and biting on the swab were grouped into “fluid-related difficulties,” and crying remained stand-alone. This was done to assess results given the small number of children reporting individual collection difficulties. We generated 95% confidence intervals and determined our alpha threshold of 0.05 for p-values.

### 2.4 Analysis of anti-TT IgG concentration in OF and serum

The multivariate regression analysis described above was extended to evaluate risk factors for low anti-TT IgG concentrations in OF. The number of pentavalent doses were consistently controlled for in all models of anti-TT IgG in OF or serum due to its clear causal effect on anti-TT IgG concentration. The best model was identified by minimizing the AIC and BIC, and maximizing the adjusted R^2^.

Given that anti-TT IgG in OF served as a proxy for anti-TT IgG in serum, risk factors affecting the relationship between the two were assessed. The analysis was stratified as below the median total IgG concentration (referred to as “low”) or above the median (referred to as “high”). Leave-one-out cross-validation (LOOCV) was performed, comparing the root-mean squared errors (RMSE) and the mean absolute errors (MAE) to identify the best fitting model for prediction of anti-TT IgG in serum. An interaction model was also subsequently generated between the category of total IgG and continuous anti-TT IgG in OF. Using this interaction model to perform a prediction analysis. Under ideal circumstances, the OF would perfectly reflect serum IgG, therefore the prediction model was also visually compared to the Y = X reference.

### 2.5 Ethics

Ethical approval was granted by the Kenya Medical Research Institute (KEMRI) Ethics Review Committee (SSC protocol #2855)**.** Johns Hopkins University Bloomberg School of Public Health deferred ethical oversight to KEMRI.

## 3 Results

### 3.1 Description of study population

Of the 445 selected for enrollment, 267 children had both an OF and serum specimen for the analytic sample (Table 1). Fifty-two percent were female and the median age was 13.7 months (range: 12.1–15.3). The majority of children were born in a health facility (66%) and most lived less than 30 min from a clinic (57%) (Table 1). Ninety-three percent of children received the three-dose pentavalent vaccination series, 8 children received two doses, 6 received one dose, and 5 children reported no pentavalent doses.

**Table 1:** Characteristics of Study Population.

	Participants N = 267
**Sociodemographic characteristics & vaccination status**	
Age (months) – median [IQR]	13.7 [12.9, 14.6]
Female, n (%)	139 (52)
Height for age z-score (HAZ)—mean (SD)	−1.51 (1.16)
Weight for age z-score (WAZ)—mean (SD)	−0.75 (1.31)
Wealth Quintile, n (%)	
Quintile 1	54 (20.4)
Quintile 2	58 (21.9)
Quintile 3	47 (17.7)
Quintile 4	48 (18.1)
Quintile 5	58 (21.9)
Number of ANC visits during last pregnancy—mean (SD)	3.3 (1.5)
Delivery in health facility, n (%)	175 (66)
Caregiver age (years)— median [IQR]	27 [23], [32]
Years of caregiver’s education— median [IQR]	8 [7], [10]
Time to clinic more than 30 min (%)	113 (43)
Number of pentavalent vaccinations received, n (%)^1^	
0	5 (2)
1	6 (2)
2	8 (3)
3	248 (93)
**Health characteristics of child, n (%)**	
Runny nose	102 (38)
Congestion	16 (6)
Dry mouth	15 (6)
Number of erupted teeth— mean (SD)	7.1 (2.9)
**Oral fluid collection difficulties, n (%)**	
Crying	226 (85)
Dry mouth	32 (12)
Sucked on swab	30 (11)
Bit swab	118 (44)
Gagged on swab	29 (11)
Food in mouth/on swab	4 (2)
Sores in mouth	3 (1)
Blood in specimen	5 (2)
At least one oral fluid collection problem	261 (98)
**Laboratory Collected**	
Oral fluid volume (μL)—mean (range)	583.1 (11–1620)
Total IgG (mg/L)—mean (range)	104.8 (5.7–1577.6)

Note: SD: standard deviation; IQR: interquartile range; HAZ: height-for-age z-score; WAZ: weight-for-age z-score; ANC: antenatal care. HAZ and WAZ calculated according to 2006 WHO Child Growth Standards.

Missing responses for 2 children for ANC visits, location of delivery, travel time to clinic, caregiver age, caregiver education; 13 for weight-for-age z-score and 3 for the number of teeth.

1 Pentavalent vaccination status was determined by having at least two records (vaccination clinic administrative data, child vaccination card, and caregiver recall) in concordance. Unvaccinated children were defined by lack of vaccination card documentation, lack of vaccination clinic record for the child, and caregiver recall that the child received no vaccinations

Forty-five percent of children had at least one health concern at the time of OF collection, primarily runny nose (38% of all children). Data collectors were able to swab all children for 1.5 min in accordance with Oracol protocols. They reported at least one OF specimen collection problem for 98% of children. Crying during collection was most frequently reported (85%) followed by 53% of children who either bit, gagged or sucked the swab. Mean total OF volume was 583 μL (range: 11–1620) from two swabs. Volume varied substantially but 97% of children produced at least 100 μL of OF from the two swabs.

### 3.2 Risk factors for total IgG concentration in OF

Demographic, socioeconomic and healthcare access were not associated with total IgG concentrations in bivariate or multivariate models (Table 2). Two laboratory-collected variables, OF volume and blood in the specimen, were positively associated with total IgG. Several specimen collection problems observed in the field were also associated with total IgG concentration in bivariate analyses: sores in the child’s mouth, blood in the specimen and crying had significant and positive associations and sucking on the swab and the child’s weight-for-age Z score were associated with lower total IgG concentration.

**Table 2:** Unadjusted and adjusted models of risk factors for low total IgG concentration (mg/L) in oral fluid.

Outcome: Total IgG in oral fluid	Unadjusted bivariate regression geometric mean (GM) ratio (95% CI)	Model 1: Laboratory adjustments only GM ratio (95% CI)	Model 2: Field-based adjustments only GM ratio (95% CI)	Model 3: Laboratory and field-based adjustments GM ratio (95% CI)
Oral fluid volume, per 100μL	**0.84 (0.81–0.88)**	**0.85 (0.82–0.88)**	—	**0.87 (0.84–0.90)**
Blood in specimen	**3.72 (1.61–8.59)**	**2.47 (1.16–5.24)**	**3.06 (1.41–6.64)**	**6.85 (2.77–16.92)**
Sores in mouth	**9.69 (1.35–28.02)**	—	**7.93 (2.87–21.89)**	**2.37 (1.17–4.77)**
Crying	**2.27 (1.67–3.08)**	—	**1.63 (1.15–2.31)**	**1.51 (1.12–2.03)**
Sucked on swab	**0.47 (0.33–0.67)**	—	**0.68 (0.46–1.00)**	**0.61 (0.44–0.85)**
Dry mouth	**1.50 (1.06–2.14)**	—	1.35 (0.96**–**1.90)	—
Bit swab	**0.79 (0.62–0.99)**	—	0.91 (0.73 **–** 1.13)	—
Child WAZ	**0.83 (0.76–0.91)**	—	0.92 (0.84**–**1.00)	—
Child HAZ	0.94 (0.85 **–** 1.03)	—	—	—
Gagged on swab	1.22 (0.8**4 –** 1.76)	—	—	—
Food in mouth	0.56 (0.22**–**1.45)	—	—	—
Number of teeth erupted	1.00 (1.00**–**1.00)	—	—	—
Congestion	1.03 (0.63**–**1.68)	—	—	—
Runny nose	1.16 (0.92**–**1.47)	—	—	—
SES (Wealth score)				
Quintile 1	*Ref*			—
Quintile 2	1.23 (0.87**–**1.75)	—	—	—
Quintile 3	1.00 (0.69**–**1.45)	—	—	—
Quintile 4	0.79 (0.55**–**1.15)	—	—	—
Quintile 5	0.71 (0.50**–**1.01)	—	—	—
Number of ANC visits	0.95 (0.88**–**1.02)	—	—	—
Female	0.92 (0.73**–**1.16)	—	—	—
Time to clinic	1.03 (0.81**–**1.30)	—	—	—
Region (Gem)	1.34 (0.98**–**1.84)	—	—	—
Adjusted R^2^	0.19	0.23	0.20	0.34
AIC (BIC)	693.84 (701.06)	667.64 (678.40)	656.95 (685.25)	631.43 (652.96)

GM Ratio: Geometric Mean Ratio; AIC: Akaike’s Information Criterion; BIC: Bayesian Information Criterion; HAZ: height-for-age Z score; WAZ: weight-for-age Z score.

*Note: Bivariate adjusted R^2^, AIC, and BIC reflect adjusted regression of oral fluid volume only on log-transformed total IgG. Statistically significant p-values are reported in bold. Results are reported as geometric mean ratios of total IgG. Statistical significance was defined as p-value < 0.05. Only statistically significant oral fluid collection difficulties were included in Model 1 without oral fluid volume, and with oral fluid volume in Model 2. Excluded covariates represented as “—“.

Models using only laboratory variables or only field variables explained 23% and 20% of the variation in total IgG, respectively. The best fit model used laboratory and field-based variables and 34% of the variation in total IgG concentration was explained by OF volume, blood in the specimen and three specimen collection variables (Model 3, Table 2). For every 100 μL of additional OF volume collected, the geometric mean concentration (GMC) of total IgG was approximately 13% lower (95% CI: 0.84–0.90). Despite only a small subset of children reporting these difficulties, blood in the specimen and sores in the mouth had the greatest effect sizes with 6.85 (95% CI: 2.77–16.92) and 2.37 (95% CI: 1.17–4.77) times higher total IgG GMC, respectively. Similar results were observed in a sensitivity analysis where sores in the mouth and blood in specimen were combined (Supplementary Table 1).

### 3.3 Risk factors for lower anti-tetanus toxoid IgG concentration in OF

To evaluate if specimen quality indicators were associated with concentrations of specific IgG in OF, multivariate regression models were run with anti-TT IgG concentration in OF as the outcome and total IgG as a predictor. Neither field-collected variables nor OF volume were significant factors after adjusting for total IgG concentration and vaccination history (Model 1 and 2, Table 3). The most parsimonious model showed that an expected 2.9% increase in geometric mean of anti-TT IgG in OF for every 10% increase in total IgG concentration (because both anti-TT in IgG and total IgG were log-transformed in the model, 1.10^(beta)^ = 1.10^(log 1.99)^ = 1.029) controlling for other variables (Table 3, Model 3).

**Table 3:** Regression models for anti-TT IgG concentration (IU/mL) in oral fluid and field- and laboratory-collected variables.

Outcome: Log anti-TT IgG in oral fluid	Unadjusted bivariate regression GM ratio (95% CI)	Model 1: Field-collected variables & total IgG GM ratio (95% CI)	Model 2: Laboratory only GM ratio (95% CI)	Model 3: Best fit model GM ratio (95% CI)
Total IgG (mg/L, log)	**1.86 (1.63–2.11)**	**2.03 (1.79–2.31)**	**1.98 (1.74–2.25)**	**1.99 (1.78–2.24)**
Number of pentavalent doses†	**2.02 (1.56–2.61)**	**2.39 (1.92–2.97)**	**2.44 (1.97–3.01)**	**2.44 (1.98–3.01)**
Oral fluid volume, per 100μL	**0.90 (0.85–0.95)**	—	**0.99 (0.95–1.04)**	—
Crying	**1.95 (1.33–2.86)**	1.00 (0.71**–**1.42)	—	—
Sucked on swab	**0.55 (0.36–0.86)**	0.98 (0.66**–**1.45)	—	—
Mouth sores	**4.26 (1.13–16.06)**	0.75 (0.24**–**2.23)	—	—
Blood in specimen	0.80 (0.28**–**2.28)	0.57 (0.25**–**1.31)	—	—
Adj R^2^*	0.25*	0.40	0.40	0.41
AIC (BIC)*	765.89 (773.07)	711.81 (736.92)	707.79 (722.14)	705.87 (716.63)

GM Ratio: Geometric Mean Ratio; AIC: Akaike’s Information Criterion; BIC: Bayesian Information Criterion. **Bold** indicates statistical significance (p < 0.05)

Note: Outcome variable and total IgG are log_10_ transformed. Exponentiated coefficients reported here represent the geometric mean ratio of anti-TT IgG comparing a unit increase in the covariate to no increase in the non-log transformed covariates. Excluded covariates for each model represented as “—“.

* Adjusted R^2^ and AIC (BIC) refer to the bivariate regression of log transformed total IgG on anti-TT IgG in oral fluid.

† Given the causal relationship between receipt of pentavalent vaccination and anti-TT IgG, the number of pentavalent doses was controlled for across all models. Pentavalent vaccine includes antigens to tetanus, diphtheria, pertussis, hepatitis B, and *Haemophilus influenzae* type B.

### 3.4 Prediction of serum anti-TT IgG concentration with OF

Anti-TT IgG in OF was compared to circulating levels in serum and, as the single predictor, had an adjusted R^2^ of 0.41. The median ratio of anti-TT IgG in OF to serum was 1/53 (IQR: 1/98, 1/27). Because the association was different when stratifying by total IgG concentration, above or below the median, stratified models and an interaction model are presented (Table 4, Supplementary Table 2). In the final stratified models, 68% of the variation in anti-TT IgG in serum was explained in the below-median strata and 74% of the variation was explained in the above-median strata using only four predictors: total IgG concentration, anti-TT IgG, oral fluid volume, and pentavalent vaccine doses (Table 4). This was also reflected in the interaction model, which had a statistically significant interaction term between total IgG concentration and anti-TT IgG. The most parsimonious interaction model explained 72% of the variance in serum anti-TT IgG by these four predictors alone (Supplementary Table 2). The predicted anti-TT IgG concentrations in OF from the interaction model highly correlated with serum concentrations (Fig. 2). The line of best fit for the prediction showed better adherence to the Y = X reference than the unadjusted fitted line between OF anti-TT IgG and that in serum.

**Table 4:** Stratified linear regression models by low and high total IgG for anti-TT IgG (IU/mL) in serum.

Model 1: Low Total IgG Stratum (less than median of specimen concentrations, <56.15 mg/L)			
Outcome: Anti-TT IgG in serum	Unadjusted model GM Ratio (95% CI)	Adjusted model GM Ratio (95% CI)	Final adjusted model GM Ratio (95% CI)
Oral fluid anti-TT IgG (IU/mL, log)	**3.33 (2.77–4.02)**	**3.46 (2.89–4.13)**	**2.81 (2.39–3.31)**
Oral fluid volume, 100μL	**—**	**1.11 (1.04–1.18)**	**1.09 (1.03–1.15)**
Number of pentavalent doses	**—**	**—**	**3.36 (2.35–4.82)**
RMSE	0.98	0.95	0.85
MAE†	0.70	0.66	0.62
Adj R^2^	0.55	0.58	0.68

**Model 2: High Total IgG Stratum** (greater than median of specimen concentrations, ≥56.15mg/L)			
**Outcome: Anti-TT IgG in serum**	**Unadjusted model** GM Ratio (95% CI)	**Adjusted model** GM Ratio (95% CI)	**Final adjusted model** GM Ratio (95% CI)
Oral fluid anti-TT IgG (IU/mL, log)	**2.58 (2.22–2.99)**	**2.61 (2.25–3.01)**	**2.07 (1.83–2.34)**
Oral fluid volume, 100μL	**—**	**1.10 (1.01–1.20)**	**1.06 (1.00–1.13)**
Number of pentavalent doses	**—**	**—**	**3.31 (2.60–4.26)**
RMSE	1.09	1.08	0.84
MAE	0.78	0.77	0.62
Adj R^2^	0.55	0.56	0.74

GM Ratio: Geometric Mean Ratio; AIC: Akaike’s Information Criterion; BIC: Bayesian Information Criterion; RMSE: root-mean-square error; MAE: mean absolute error.

Note: Unadjusted and adjusted models of anti-TT IgG concentration in oral fluid on anti-TT IgG concentration in serum stratified by low/high total IgG. Excluded covariates for each model represented as “—“. The final adjusted model used in each strata was: *Anti-TT IgG in Serum = α + β(anti-TT IgG in OF) + β(OF Volume) + β(Pentavalent Doses)*

† MAE calculated by leave-one-out cross validation.

## 4 Discussion

This study showed that the concentration of specific IgG antibodies detected in OF are affected by the characteristics and quality of the OF specimen. We identified several specimen quality variables measured in the field or laboratory that can be used to adjust IgG antibody concentrations in OF to better predict circulating concentrations in serum. Two specimen quality variables which are both measured in the laboratory, total IgG concentration and OF volume, generated more accurate models than variables collected in the field. OF can be a good proxy for circulating antibody concentrations when total IgG concentration, OF volume, and vaccination history are also measured and adjusted for in post-hoc analyses.

OF volume and blood in the specimen were strongly associated with total IgG concentration, which is an indicator of specimen quality. Specimen volume was inversely associated with total IgG, suggesting that a high-volume specimen is not necessarily a better quality specimen. Given that OF antibody levels was measured using an indirect EIA assay, measuring and adjusting for specimen volume is reasonable. In contrast, capture EIAs have been recommended for use with OF because they measure the proportion of total IgG that is antigen specific and results are less susceptible to variability in specimen volume than other methods [35]. Blood in the specimen and mouth sores were associated higher total IgG concentrations, as would be expected from a transudated fluid [18], [38]. Blood contamination was assessed by judging the color of the centrifuged OF specimen in this study, though this could also be measured by transferrin or albumin to adjust for in models [39], [40].

**Fig. 1:**
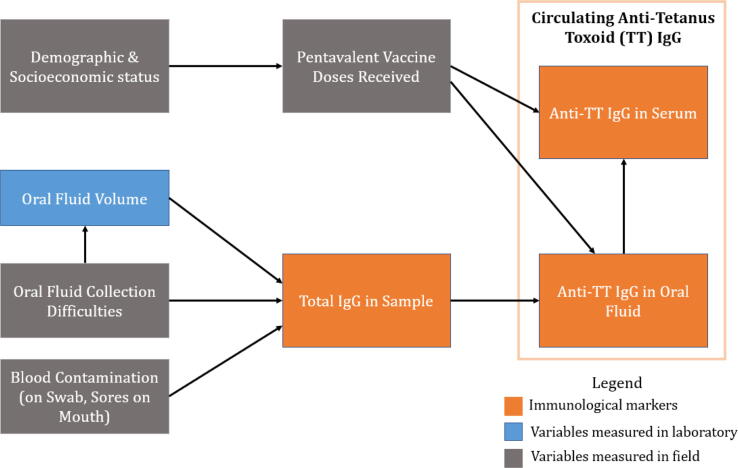
Directed acyclic graph (DAG) of risk factors contributing to poor quality oral fluid specimens and discrepancies in antibody detection between serum and oral fluid.

When the model was extended to specific IgG in OF with total IgG as a predictor, OF volume and blood in the specimen were no longer significant, suggesting that total IgG mediates the effect of collection difficulties. Full mediation is consistent with the DAG given there is no theorized biological reason why, for example, crying would result in lower anti-TT IgG outside the total IgG pathway (Fig. 1). Other studies have observed similar relationships between total and specific IgG: one study found a positive correlation between these in OF [40], and another reported increased risk of misclassification for specimens with low total IgG [28]. Because it is not possible to assess the quality of an OF specimen in the field, several studies measure total IgG and exclude very low concentration specimens [3], [41], but this does not account for discrepancies in low and moderate concentration specimens. This study recommends adjusting all specific IgG indirect ELISA results in oral fluid for total IgG.

**Fig. 2:**
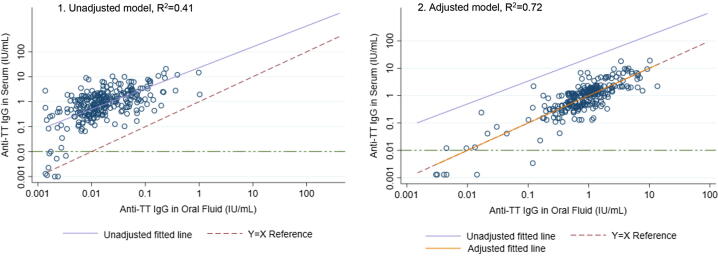
Comparison of unadjusted and predicted fit with interaction model of anti-TT IgG in oral fluid and serum. Note: Panel 1 depicts the unadjusted values of anti-TT IgG in oral fluid vs. serum as dark blue points and unadjusted fitted line in blue, compared to the Y = X reference line, represented as a red dashed line. Panel 2 depicts the same unadjusted fitted line and the adjusted values and fitted line in solid orange after adjustment with interaction model. The equation for the adjusted model was: Anti-TT IgG in Serum = α + β(anti-TT IgG in OF) + β(Total IgG in OF) + β(anti-TT IgG in OF*Total IgG in OF) + β(OF Volume) + β(Pentavalent Doses). Green horizontal line at 0.01 IU/mL represents cutoff of serological protection for the assay. (For interpretation of the references to color in this figure legend, the reader is referred to the web version of this article.)

Ideally, OF IgG levels would be a window into circulating levels, and anti-TT IgG concentration in OF would correlate perfectly with serum anti-TT IgG. This study demonstrated that 72% of variation in serum anti-TT IgG could be explained by measuring concentrations in OF and accounting for OF volume, the number of pentavalent doses received and effect modification by total IgG. This is similar, but with reduced correlation, to a prior study utilizing this OF testing procedure, which found a correlation of >90% when using linear regression. [27] However, this study was conducted at 12 months of age, while the study by Tapia et al., used data from children as young as 2 months, to adults. Among their participants age 4 months and above, all of the particpants had received at least one dose of DTP vaccine, while this study specifically sampled for those with no doses as well. These key differences in study samples may partially explain the differences between our adjusted R^2^ estimates, though we show a high level of correlation overall, suggesting this method, with adjustment, may still lead to overall similar results despite population differences. While OF volume was significant and improved model fit in certain analyses, the effects were relatively small and varied by analysis. While a minimum specimen volume is essential for testing, OF volume was a less influential predictor of antigen-specific IgG in serum than total IgG concentration.

The lower concentration of IgG antibodies in OF is a risk for using less invasive specimens to estimate circulating IgG levels, especially for antibodies in low concentration in blood. We found that the median concentration of anti-TT IgG in OF was 1/53 of levels detected in serum, which is substantially higher than other studies [13] and may be related to the specimen collection and processing method. In this study, OF volume was measured and diluted at 1:1 ratio in preservation buffer. Other studies dilute in a constant buffer volume, such as 1 mL, which disproportionately dilutes small volume samples [3], [42]. Preservation of undiluted samples has generated high quality results, likely attributable to the robustness of IgG antibodies to proteases, temperature and other degradation. In this study, 55% of the variability of circulating anti-TT IgG was explained by levels in OF alone (Table 4). Similar correlation between serum and saliva are observed for malaria (63.0–64.0% [39]), rubella (43.4–56.6% [43]) and toxoplasmosis (45.6–59.8% [38]). Like Riis et al. [44] and Facente et al. [45], a post hoc adjustment for OF collection problems improved predictive accuracy. Yet 28% of variability of serum remained unexplained in the model, which may be due to inherent variability in the specimen [18] and highlights the importance of swab type and protocols for collection, processing and storage specimens [17]. Additionally, while testing sera at a different facility than OF may introduce measurement differences based on formats or reagents used by the respective laboratories, using the Statens Serum Institut double ELISA allowed for greater accuracy in the serum measurements than we would obtain by using the same indirect ELISA as the OF [36]. This allowed us to obtain a gold standard with higher validity to compare to our OF results to reduce measurement bias when estimating our results.

Our study had limitations including the lack of external validation dataset for our prediction model and the small number of un- and undervaccinated subgroups. While the predicted anti-TT IgG concentration showed high correlation with serum, further research is needed in other populations to test its validity and to further elucidate a meaningful cutoff with total IgG levels. Ideally, such a dataset would include individuals across the range of anti-TT IgG concentrations. High pentavalent vaccination coverage in this study and overall in Kenya limited inferences about children with little or no circulating anti-TT IgG [5]. To assess the generalizability of these post hoc adjustments, additional analyses comparing OF and blood are needed.

Previous studies have shown variable accuracy of OF assays for measures of IgG antibodies [38], [39], [40]. This study serves as a careful characterization of a broad range of specimen collection factors that may explain those variable results. To our knowledge, no study has previously reported the prevalence of OF collection difficulties encountered by field staff and their impact on serology. These results show that OF collection problems reduce the correlation between OF and serum IgG levels and that the discrepancies can largely be controlled for by the processing laboratory through adjustment for the number of pentavalent doses, total IgG concentration, and specimen volume. We recommend researchers using oral fluid for seroepidemiology also measure these variables, and consider adjusting for them in analyses.

## Declaration of Competing Interest

The authors declare the following financial interests/personal relationships which may be considered as potential competing interests: [Dr. Hani Kim was employed by Johns Hopkins University at the time of her involvement with the study during study set-up and data collection. Since her involvement, she has transitioned to a role at the Bill and Melinda Gates Foundation. All authors report no conflict of interest.].
