# Supplementary material for: Adjustments for oral fluid quality and collection methods improve prediction of circulating tetanus antitoxin: Approaches for correcting antibody concentrations detected in a non-invasive specimen
Source: Vaccine. 2021 Jan 8;39(2):423–30. doi: 10.1016/j.vaccine.2020.11.027 (PMC7805266; doi:10.1016/j.vaccine.2020.11.027)

**Appendix.**

**Supplementary Table S1.** **Sensitivity analysis of grouping oral fluid collection problems: Unadjusted and adjusted models of risk factors for low total IgG concentration in oral fluid**

|  | **Unadjusted bivariate regression**  GM^‡^ ratio (95% CI) | **Model 1:**  **Laboratory adjustments only**  GM ratio (95% CI) | **Model 2:**  **Field-based adjustments only**  GM ratio (95% CI) | **Model 3:**  **Laboratory and field-based adjustments**  GM ratio (95% CI) |
| --- | --- | --- | --- | --- |
| Oral fluid volume, per 100 $\mu L$ | **0.84 (0.81** **–** **0.88)** | 0.85 (0.82 – 0.88) | — | **0.86 (0.83 – 0.90)** |
| Blood-related collection difficulties* | **5.46 (2.86 – 10.43)** | **3.55 (1.97** – **6.40)** | **4.29 (2.28** – **8.10)** | **0.34 (1.92** – **6.06)** |
| Crying | **2.27 (1.67 – 3.08)** | — | **1.98 (1.44** – **2.72)** | **1.77 (1.33 – 2.36)** |
| Other collection difficulties^†^ | 0.87 (0.69 – 1.10) | — | 1.04 (0.83 – 1.30) | **1.07 (0.87 – 1.31)** |
| AIC | 712.03 | 655.30 | 690.63 | **640.13** |
| BIC | 658.06 | 666.06 | 704.98 | **658.06** |
| Adj $R^{2}$ | 0.09 | 0.27 | 0.16 | 0.31 |

GM ratio: Geometric mean ratio; CI: Confidence Interval

*Blood-related collection difficulties include: Blood in sample, sores in mouth, inflammation in mouth

^†^ Other collection difficulties include: Food in mouth, gagged on swab, sucking on swab, dry mouth

**Supplementary Table S2. Extended risk factor analysis of interaction models of total IgG and oral fluid anti-TT IgG for prediction of circulating anti-TT IgG in serum**

|  | **Model 1:**  **Saturated interaction model**  GM Ratio (95% CI) ^†^ | **Model 2:**  **Interaction model with sociodemographic covariates**  GM Ratio (95% CI) | **Model 3:**  **Interaction model with oral fluid collection difficulties**  GM Ratio (95% CI) | **Model 4:**  **Final interaction model coefficients**  GM Ratio (95% CI) |
| --- | --- | --- | --- | --- |
| Oral fluid volume, per 100 $\mu L$ | **1.07 (1.02 – 1.12)** | **1.07 (1.03 – 1.13)** | **1.07 (1.02 – 1.11)** | **1.07 (1.03** – **1.12)** |
| Number of pentavalent doses* | **3.64 (2.91 – 4.55)** | **3.59 (2.88 – 4.46)** | **3.65 (3.00 – 4.50)** | **3.65 (2.97 – 4.48)** |
| Oral fluid anti-TT IgG (IU/mL, log) ^a^ | **2.91 (2.44 – 3.48)** | **2.82 (2.37 – 3.36)** | **2.89 (2.45 – 3.42)** | **2.82 (2.40 – 3.33)** |
| Total IgG, above median cutoff ^b^ | **0.11 (0.05 – 0.28)** | **0.12 (0.05 – 0.29)** | **0.11 (0.05 – 0.26)** | **0.12 (0.05 – 0.27)** |
| Interaction term – total IgG & oral fluid anti-TT IgG ^c^ | **0.70 (0.57 – 0.86)** | **0.72 (0.58 – 0.88)** | **0.70 (0.58 – 0.86)** | **0.71 (0.59 – 0.87)** |
| Blood in specimen | 1.04 (0.49 – 1.09) | -- | 1.01 (0.49 – 2.07) | -- |
| Sores in mouth | 0.50 (0.49 – 2.22) | -- | 0.47 (0.19 – 1.19) | -- |
| Crying | 0.86 (0.62 – 1.21) | -- | 0.89 (0.65 – 1.22) | -- |
| Sucked on swab | 1.22 (0.83 – 1.80) | -- | 1.23 (0.86 – 1.75) | -- |
| Child WAZ | 0.99 (0.90 – 1.09) | 1.02 (0.93 – 1.12) | -- | -- |
| Child HAZ | 0.97 (0.87 – 1.08) | 0.95 (0.86 – 1.06) | -- | -- |
| Number of teeth erupted | 1.01 (0.97 – 1.05) | 1.01 (0.97 – 1.05) | -- | -- |
| SES (Wealth Score) |  |  |  |  |
| Quintile 1 | *Ref* | *Ref* |  |  |
| Quintile 2 | 0.98 (0.71 – 1.36) | 0.99 (0.72 – 1.37) | -- | -- |
| Quintile 3 | 1.08 (0.77 – 1.51) | 1.08 (0.77 – 1.51) | -- | -- |
| Quintile 4 | 1.04 (0.74 – 1.47) | 1.05 (0.75 – 1.48) | -- | -- |
| Quintile 5 | 1.04 (0.75 – 1.44) | 1.05 (0.76 – 1.44) | -- | -- |
| Number of ANC visits | 0.98 (0.91 – 1.05) | 0.98 (0.91 – 1.05) | -- | -- |
| Female | 1.05 (0.84 – 1.30) | 1.05 (0.85 – 1.29) | -- | -- |
| Time to clinic | 0.94 (0.76 – 1.16) | 0.93 (0.76 – 1.15) | -- | -- |
| Region (Gem) | 0.93 (0.70 – 1.24) | 0.89 (0.80 – 1.25) | -- | -- |
| Congestion | 1.40 (0.90 – 2.15) | 1.40 (0.91 – 2.17) | -- | -- |
| Runny nose | 1.00 (0.80 – 1.25) | 1.00 (0.80 – 1.25) | -- | -- |
| Adj R^2^ | 0.72 | 0.74 | 0.73 | 0.72 |
| AIC | 622.63 | 619.60 | 610.54 | 607.89 |
| BIC | 703.53 | 686.43 | 645.92 | 629.12 |
| LRT compared to Model 4 | LR Chi^2^= 9.80  p=0.91 | LR Chi^2^= 4.83  p=0.98 | LR Chi^2^= 5.35  p=0.25 | -- |

Note: GM ratio: Geometric mean ratio; AIC – Akaike’s Information Criterion, BIC – Bayesian Information Criterion; LRT – Likelihood ratio test for nested models; CI: Confidence Interval; HAZ: height-for-age Z score; WAZ: weight-for-age Z score.

* Given the causal relationship between receipt of pentavalent vaccination and anti-TT IgG, the number of pentavalent doses was controlled for across all models. Pentavalent vaccine includes antigens to tetanus, diphtheria, pertussis, hepatitis B, and *Haemophilus influenzae* type B.

^a^ Anti-TT IgG in serum and anti-TT IgG in oral fluid are log­_10_ transformed.

^b^ Total IgG is a binary variable dichotomized as above and below the mean total IgG value.

^c^ The interaction term represents the multiplicative interaction between being above the total IgG mean and a 1 IU/mL increase in oral fluid anti-TT IgG (log-transformed).

Exponentiated coefficients reported here represent the geometric mean ratio of anti-TT IgG in serum comparing a unit increase in the covariate to no increase in the non-log transformed covariates. Excluded covariates for each model represented as “—“.

**Supplementary Figure S3.** Histogram of total IgG concentration ($\mu$g/mL) on the logarithmic scale.


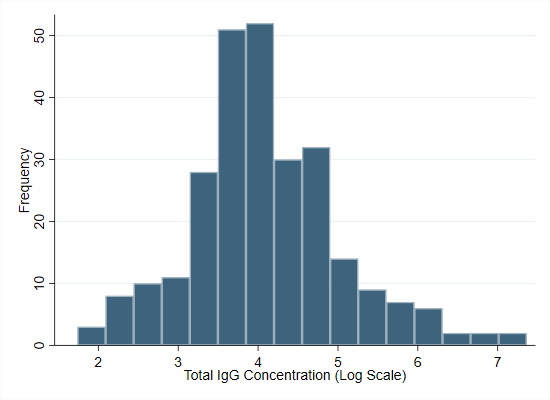


**Supplementary Figure S4.** Histogram of total oral fluid volume in samples.


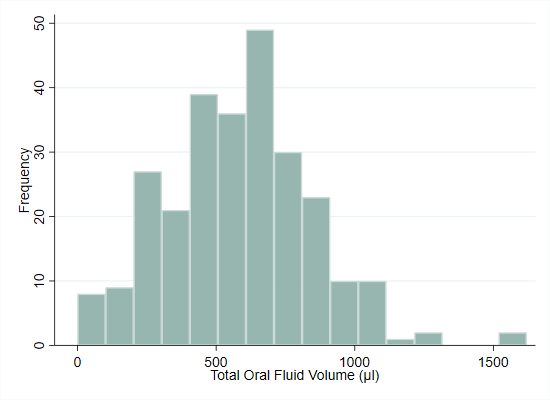

Supplement: Supplementary data 1 [file mmc1.docx]
